# Microstructural characterization and inductively coupled plasma-reactive ion etching resistance of Y_2_O_3_–Y_4_Al_2_O_9_ composite under CF_4_/Ar/O_2_ mixed gas conditions

**DOI:** 10.1038/s41598-024-57697-5

**Published:** 2024-03-25

**Authors:** Ho Jin Ma, Seonghyeon Kim, Ha-Neul Kim, Mi-Ju Kim, Jae-Woong Ko, Jae-Wook Lee, Jung-Hyung Kim, Hyo-Chang Lee, Young-Jo Park

**Affiliations:** 1 Department of Engineering Ceramics, Korea Institute of Materials Science, Changwon, 51508 Republic of Korea; 2 Semiconductor Integrated Metrology Team, Korea Research Institute of Standards and Science, Daejeon, 34113 Republic of Korea; 3 Department of Semiconductor Science, Engineering and Technology, Korea Aerospace University, Goyang, 10540 Republic of Korea; 4 School of Electronics and Information Engineering, Korea Aerospace University, Goyang, 10540 Republic of Korea

**Keywords:** Plasma etching, Y_2_O_3_-YAM composite, Microstructure, Etching resistance, Semiconductor manufacturing, Ceramics, Composites

## Abstract

In the semiconductor manufacturing process, when conducting inductively coupled plasma-reactive ion etching in challenging environments, both wafers and the ceramic components comprising the chamber’s interior can be influenced by plasma attack. When ceramic components are exposed to long-term plasma environments, the eroded components must be replaced. Furthermore, non-volatile reactants can form and settle on semiconductor chips, acting as contaminants and reducing semiconductor production yield. Therefore, for semiconductor processing equipment parts to be utilized, it is necessary that they exhibit minimized generation of contaminant particles and not deviate significantly from the composition of conventionally used Al_2_O_3_ and Y_2_O_3_; part must also last long in various physicochemical etching environment. Herein, we investigate the plasma etching behavior of Y_2_O_3_–Y_4_Al_2_O_9_ (YAM) composites with a variety of mixing ratios under different gas fraction conditions. The investigation revealed that the etching rates and changes in surface roughness for these materials were significantly less than those of Y_2_O_3_ materials subjected to both chemical and physical etching. Microstructure analysis was conducted to demonstrate the minimization of crater formation. Mechanical properties of the composite were also analyzed. The results show that the composite can be commercialized as next-generation ceramic component in semiconductor processing equipment applications.

## Introduction

The transition of semiconductor-based devices from 2D to 3D-NAND memory, accompanied by increased miniaturization using advanced technologies, necessitates the incorporation of high aspect ratio and line width miniaturization. Consequently, there arises a need for a diversified plasma environment in the etching process^1–3^. Inductively coupled plasma-reactive ion etching (ICP-RIE) has proved a suitable method for achieving deeper and higher aspect ratio chip etching, as it achieves high plasma density at low pressures, thereby facilitating a heightened etching rate^4^. Notably, during the etching process, the strong plasma affects both the Si wafers and the ceramic parts constituting the chamber interior, such as focus rings, confinement rings, life pins, and inner walls. These ceramic components have varying replacement cycles depending on where they are used and what materials they are made of. For instance, the confinement ring, which uses alumina, should be replaced every 300 h; the focus ring every 6 months^5^. Longer replacement intervals can elevate semiconductor production yields by eliminating the need to shut down the chamber.

Particulate contamination, originating from internal parts shaved off by physical ion sputtering and generated by chemical reactions between radicals and the surfaces of ceramics, can be produced during ICP-RIE process. Certain volatile reactants, like SiF_4_, are eliminated using a vacuum pump. On the other hand, others may become trapped within the sheath and have the potential to migrate towards the upper surface of the wafer once the plasma ceases to operate^6,7^. Given that this circumstance can result in reduced production yield of large-scale integrated circuit chips, it is imperative to carefully select and develop appropriate ceramic materials that do not generate contaminants^8–10^. Furthermore, for more sophisticated etching of semiconductor chips, processing is carried out in various gas environments such as CF_4_, CHF_3_, Cl_2_, etc.^11–13^. The fluorine and argon gas mixture has been studied and is applied for etching processes on Si-based semiconductors^14,15^. O_2_ gas can increase the yield of F atoms through its interaction with CF_4_ gas, while also preventing polymerization on surfaces exposed to the plasma. Consequently, it helps in attaining the targeted etching selectivity and feature topology^14,16^. The introduction of Ar elevates plasma density and enhances generation of F^−^ active species. Given that physicochemical plasma etching processes operate concurrently, manipulation of the plasma gas fraction enables the realization of selective etching^17^.

For decades, research has been conducted on the plasma resistance properties of various ceramic bulk and coating materials in chamber during semiconductor etching in halogen plasma gas atmosphere^18,19^. Y_2_O_3_ material has been the subject of interest and commonly utilized because of its outstanding chemical etching resistance^20–23^. Through a reaction of Y_2_O_3_ surfaces with radicals, Y_2_O_3_ can form a thick and non-volatile fluorinated layer. This layer serves as effective protection for components against ions impingement^24^. However, in conditions in which physical etching predominates and there is a high bias voltage, the fluorinated layer on the surface of the Y_2_O_3_ matrix is prone to removal. This results in its conversion into contaminant particles. Also, cracks form at the interface of the matrix and the fluorinated material^25^. In addition, for sintered Y_2_O_3_ ceramics that become densified at high sintering temperature, large grain sizes can cause large craters after etching, resulting in rougher surface roughness and large contaminant particles^26^. The hardness is low, and so improvement of mechanical properties is essential for applications in ceramic parts^27^.

We recently found that Y_2_O_3_–MgO nanocomposite material could improve the physicochemical plasma etching resistance under intense plasma conditions^28^. In the temperature range at which the two composition do not react, each composition suppressed unintended grain coarsening via Zener pinning effect and minimized the development of large craters in the microstructure^29,30^. Research has also explored Y_3_Al_5_O_12_ (YAG) ceramic as a highly promising material resistant to plasma in etching chambers^31,32^. However, for YAG, when encountering Y_2_O_3_, and depending on the mixing fraction, YAlO_3_ (YAP) phase with perovskite structure and Y_4_Al_2_O_9_ (YAM) phase with monoclinic structure can form as reactants^33,34^. Therefore, the Y_2_O_3_-YAM composition is best suited to form composites with both compositions, without additional reaction. The YAM ceramic has a moderate thermal expansion coefficient (7.51 × 10^–6^ K^−1^), high melting point (2020 °C), and low high temperature thermal conductivity (1.13 W/m/K)^35,36^. Therefore, it is anticipated that this material can be applied as a thermal barrier coating (TBC). Unlike YAG, little research has been done on the plasma etching resistance of YAM ceramics. When ceramic components used in semiconductor process equipment are completely changed from existing materials, it is difficult in practice to apply them due to the lack of reported processing stability. However, Y_2_O_3_, Al_2_O_3_, and YAG materials are all being used or developed in chambers, so they are free of this problem. In addition, these composites can be used to overcome the shortcomings of conventional Y_2_O_3_ materials.

In this study, we investigate the plasma etching characteristics of Y_2_O_3_-YAM composite across different volume fractions; material is intended for application in ceramic parts within etching equipment operating under high ICP power and bias voltage conditions. Firstly, dense Y_2_O_3_ and YAM composite sintered bodies with different volume ratios were fabricated via hot-press sintering. Next, the etching depths and surface roughness changes of Y_2_O_3_ and Y_2_O_3_-YAM composites were analyzed after physicochemical plasma etching with different mixture gas ratios. The mechanical properties of the composites and the surface microstructure after etching were analyzed. This investigation examined the capability of Y_2_O_3_-YAM composite to substitute for conventional materials to minimize production of contaminants and improve the production yield of semiconductors when ceramic components are subjected to intense plasma environments and diverse gas conditions.

## Methods

### Fabrication of Y_2_O_3_-Y_4_Al_2_O_9_ nanocomposites ceramic

Y_2_O_3_ (99.9%, Cenotec, Korea) and Y_4_Al_2_O_9_ (YAM, 99.9%, Syntech, Korea) were used as raw materials for the nanocomposites. They were mixed at 90:10, 70:30, 50:50, 30:70, and 10:90 volume ratios, and ball-milled with anhydrous alcohol (99.9%, Samchun, Korea) and zirconia grinding media with 5 mm diameter. The milling time was 12 h at 200 rpm. Specimens were named as shown in Table 1. After ball milling, slurries were dried and subjected to sieving through a 200-mesh sieve (75 $\mu\text{m}$). Subsequently, the sieved powders were consolidated via hot-press sintering. The graphite sleeves and spacers used for sintering were coated with BN spray to reduce direct contact with the powders and minimize contamination of samples by carbon. The Y_2_O_3_-YAM pellets were sintered at 1500 °C with applied pressure of 40 MPa under vacuum condition. The dwell time was fixed at 2 h; this was followed by cooling. After the hot-pressing, annealing was conducted at 1200 °C for 20 h in a box furnace under air atmosphere. The post-annealing process was carried out due to remove residual carbon and oxygen vacancies, and to alleviate residual stress. To analyze the microstructure of the produced composites by SEM, they were subjected to thermal etching at 1150 °C for 2 h.

**Table 1:** Composition ratios of Y_4_Al_2_O_9_ (YAM) and Y_2_O_3_ ceramics.

Sample name	Y_4_Al_2_O_9_(YAM) (Vol%)	Y_2_O_3_ (Vol%)
Y_2_O_3_	0	100
YAM1	10	90
YAM3	30	70
YAM5	50	50
YAM7	70	30
YAM9	90	10

### Plasma etching test

To elucidate the plasma etching characteristics of the Y_2_O_3_-YAM composite across various mixed gas ratio environments, all samples were produced with uniform dimensions of 15 mm diameter and 1 mm thickness. Additionally, for comparative analysis of plasma etching behavior, commercial c-axis sapphire and Y_2_O_3_ polycrystalline ceramics were prepared. The Y_2_O_3_ samples were sourced from FineTech Co, Ltd. (Korea); the measured relative density of the Y_2_O_3_ ceramics was 99.4%. To accurately identify post-etching changes of Y_2_O_3_ and Y_2_O_3_-YAM polycrystalline ceramics, it was necessary to reduce the initial surface roughness to a level of 5 nm or less, so the surface was polished and chemical-mechanically planarized (CMP). The polished samples then underwent partial coverage with a shadow mask composed of a nickel–cobalt alloy, manufactured through the electroforming process. This shadow mask is mechanically flexible, reusable and full-surface contactable, so that it can be applied in actual plasma etching process. Therefore, this material was utilized as the shadow mask to create an environment similar to the actual process. The selectively exposed area had a length of 6 mm and a width of 1 mm. The thickness of the employed mask was 0.05 mm. Details of the plasma etching conditions and a simple schematic illustration of the plasma etching test chamber system are presented in Table 2. For the plasma test, a 13.56 MHz power supply was connected to a planar-type antenna. The input ICP power and RF bias voltage were 1.5 kW and 600 V, respectively. Under the specified discharge conditions, the plasma was sustained in inductive mode; this is a well-established approach in industrial semiconductors^37^. Plasma tests were executed within an RF-biased inductively coupled plasma (ICP) chamber, employing a gas mixture of CF_4_, O_2_ and Ar^38^. The plasma density measured by a microwave cut-off probe, was 9 × 10^10^ cm^-3^. Exposure of all ceramic samples to the plasma environment was carried out for a duration of 1 h. The experimental pressure was 20 mTorr. To elucidate the physicochemical etching behavior of materials, the proportions of CF_4_:Ar:O_2_ gases were systemically adjusted at the following ratios: 40:10:10, 30:20:10, 20:30:10, and 10:40:10 sccm. In this study, the O_2_ flow rate was maintained at 10 sccm, with variation made solely to the CF_4_ to Ar gas ratios to control the physical and chemical etching. The plasma gas composition ratios used in this experiment are shown in Table 3.

**Table 2:** Details of plasma etching conditions.

	Conditions
Samples	Sapphire, Y_2_O_3_, Y_2_O_3_-YAM composite
ICP power (frequency)	1.5 kW (13.56 MHz)
Bias voltage (frequency)	600 V (2 MHz)
Plasma gas	CF_4_, Ar, O_2_
Pressure	20 mTorr
Etching time	60 min
Electrode distance	152 mm

**Table 3:** Mixed gas ratios for plasma etching test.

Gas conditions	CF_4_ (sccm)	Ar (sccm)	O_2_ (sccm)
1	40	10	10
2	30	20	10
3	20	30	10
4	10	40	10

### Characterization

Phase analysis of the sintered Y_2_O_3_-YAM composite ceramics was conducted utilizing an X-ray diffraction analysis equipment (XRD, D/Max 2500, Rigaku) with CuK $\alpha$ radiation, employing a scan rate of 5°/min within the range of 20° to 60°. The relative densities of all sintered samples were determined by the Archimedes method. The grain size was obtained by measuring the average line-intercept length of 150 grains. Average etching depths of sapphire, Y_2_O_3_, and Y_2_O_3_-YAM composites were assessed using a surface profiler (Tencor P-7 Stylus Profiler, KLA Co.) at three distinct positions for each specimen, employing scan length of 1 mm and scan rate of 200 Hz. Surface roughness and 3D topography of samples were further investigated through atomic force microscopy (AFM, XE-100, Park Systems), with the surface roughness (R_a_) derived from measurements over 25 × 25 $\mu\text{m}$ area. Microstructural images and EDS analyses of specimens were carried out using a field emission-scanning electron microscope (FE-SEM, JSM-7800F, JEOL). The Vickers hardness was measured using a Vickers hardness tester (HM200, Mitutoyo) with a 1 kg load applied to the unetched surface.

## Results and discussion

### Characterization of sintered Y_2_O_3_-YAM nanocomposites

**Figure 1:**
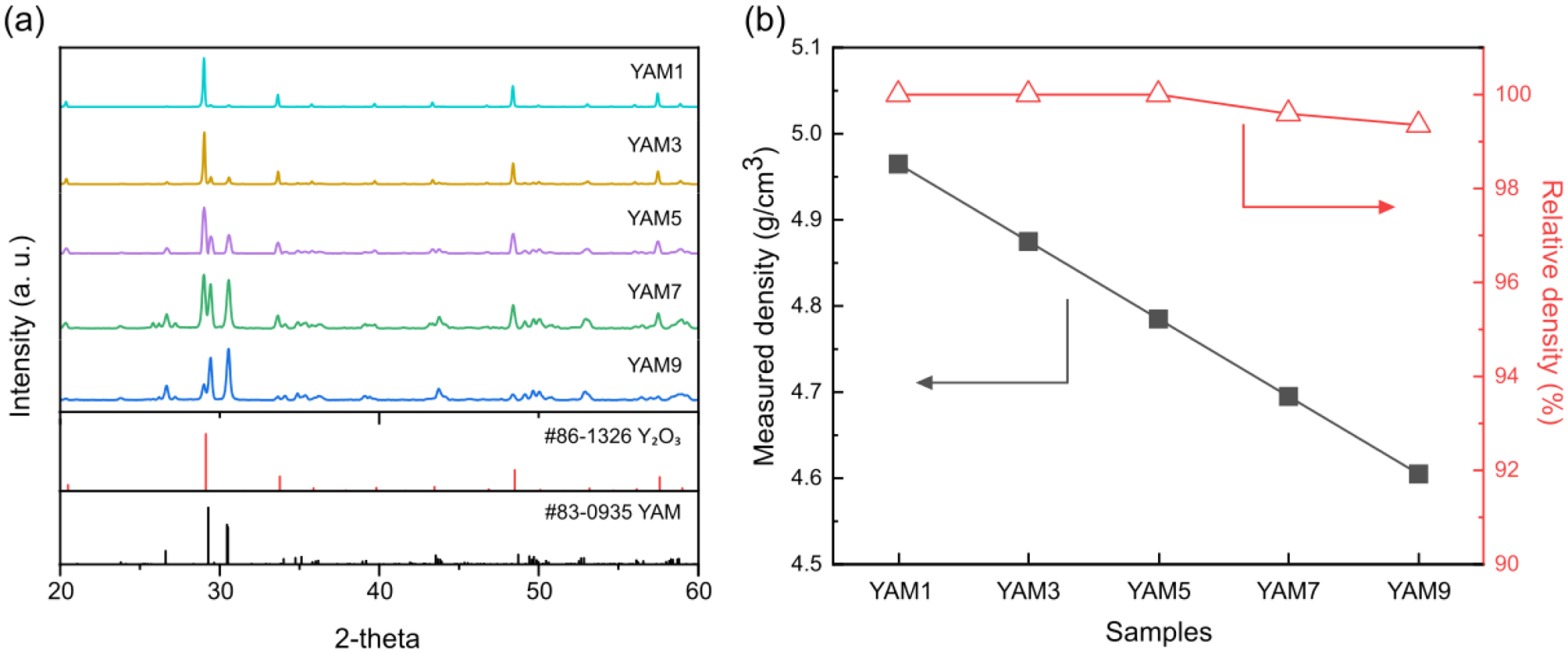
(**a**) X-ray diffraction patterns of sintered Y_2_O_3_-YAM composite ceramics with different volume ratios. (**b**) Measured and relative density of sintered Y_2_O_3_-YAM nanocomposite ceramics with different mixed ratios.

Following the blending of Y_2_O_3_ and YAM powders at varying volume ratios, the phases of the resultant composite ceramics, consolidated through hot-pressing at 1500 °C, were identified by XRD analysis, as shown in Fig. 1a. Notably, with as little as 10% YAM powder by volume, a predominantly cubic Y_2_O_3_ (#86-1326) phase was identified, accompanied by a faint monoclinic YAM (#83-0935) phase. However, the intensity of the YAM phase peak relative to Y_2_O_3_ increased as the proportion of the YAM phase increased. When Y_2_O_3_ was more than twice as abundant as Al_2_O_3_, only YAM and Y_2_O_3_ phases existed as phase diagrams in the pseudo-binary system during sintering at 1500 °C^39^. Therefore, only YAM and Y_2_O_3_ phases were detected in the composites sintered in this study, while phases such as Al_2_O_3_, Y_3_Al_5_O_12_ and YAlO_3_ were not identified.

Figure 1b shows the measured and relative densities of the Y_2_O_3_-YAM composite sintered at 1500°C. YAM has a lower density (4.56 g/cm^3^) than that of Y_2_O_3_ (5.01 g/cm^3^), so when the volume fraction ranged from 10 to 90%, the measured density exhibited a gradual decline from 4.97 to 4.61 g/cm^3^. With increased percentage of YAM phase, despite a slight decrease in relative density from nearly 100% theoretical density to 99.4%, overall, well-densified specimens with minimal residual pores were obtained. In comparison to YAM, which typically necessitates sintering temperatures as high as 1800°C and high pressure, or Y_2_O_3_ single-composition ceramics, which require a temperature of 1600°C and applied pressure, the composite of the two compositions facilitated the production of high-density specimens at lower temperatures^35,40,41^. In addition, when compounding between Y_2_O_3_ and YAG compositions, densification was difficult due to reactant formation, including YAM and YAP phases; however, in this study, by compounding Y_2_O_3_ and YAM, we were able to solve this problem and achieve high density. The process also permitted examination of the plasma resistance properties among specimens of nearly equivalent density.

**Figure 2:**
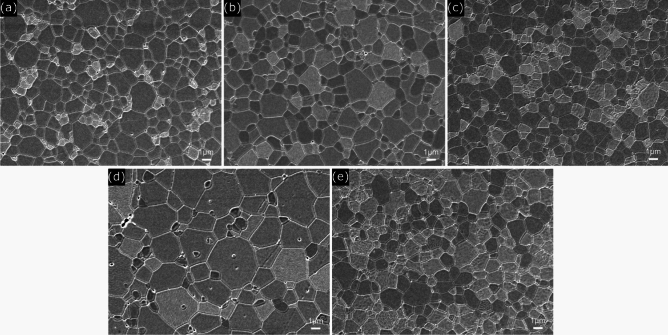
SEM microstructure images of Y_2_O_3_-YAM composites with different volume ratios after hot-pressing at 1500 °C with 40 MPa for 2 h; (**a**) YAM1, (**b**) YAM3, (**c**), YAM5, (**d**), YAM7, and (**e**) YAM9 composite ceramics.

SEM microstructural images of Y_2_O_3_-YAM composite with various volume ratios after consolidation at 1500°C for 2 h are represented in Fig. 2. Overall, dense specimens with few pores were achieved for all compositions after hot-pressing. The average grain sizes were $0.90\pm 0.53 \mu m, 1.02 \pm 0.44 \mu m, 0.86\pm 0.41 \mu m, 1.62\pm 1.02 \mu m,\text{ and }0.97\pm 0.44 \mu m$ from YAM1 to YAM9, in order, indicating an overall submicron meter size. In Fig. 2a–c, YAM1, 3, and 5 had a relatively fine grain size, with a small amount of nanopores at the triple pints, which could be the point of crater formation during plasma etching. On the other hand, the YAM7 specimen in Fig. 2d showed a relatively large grain size. It can be seen that due to the rapid grain coarsening during the sintering, pores are trapped within the grain without being able to escape. These intragranular pores are more difficult to eliminate than pores at grain boundaries or triple points.

### Plasma etching behavior of sintered Y_2_O_3_-YAM nanocomposites

**Figure 3:**
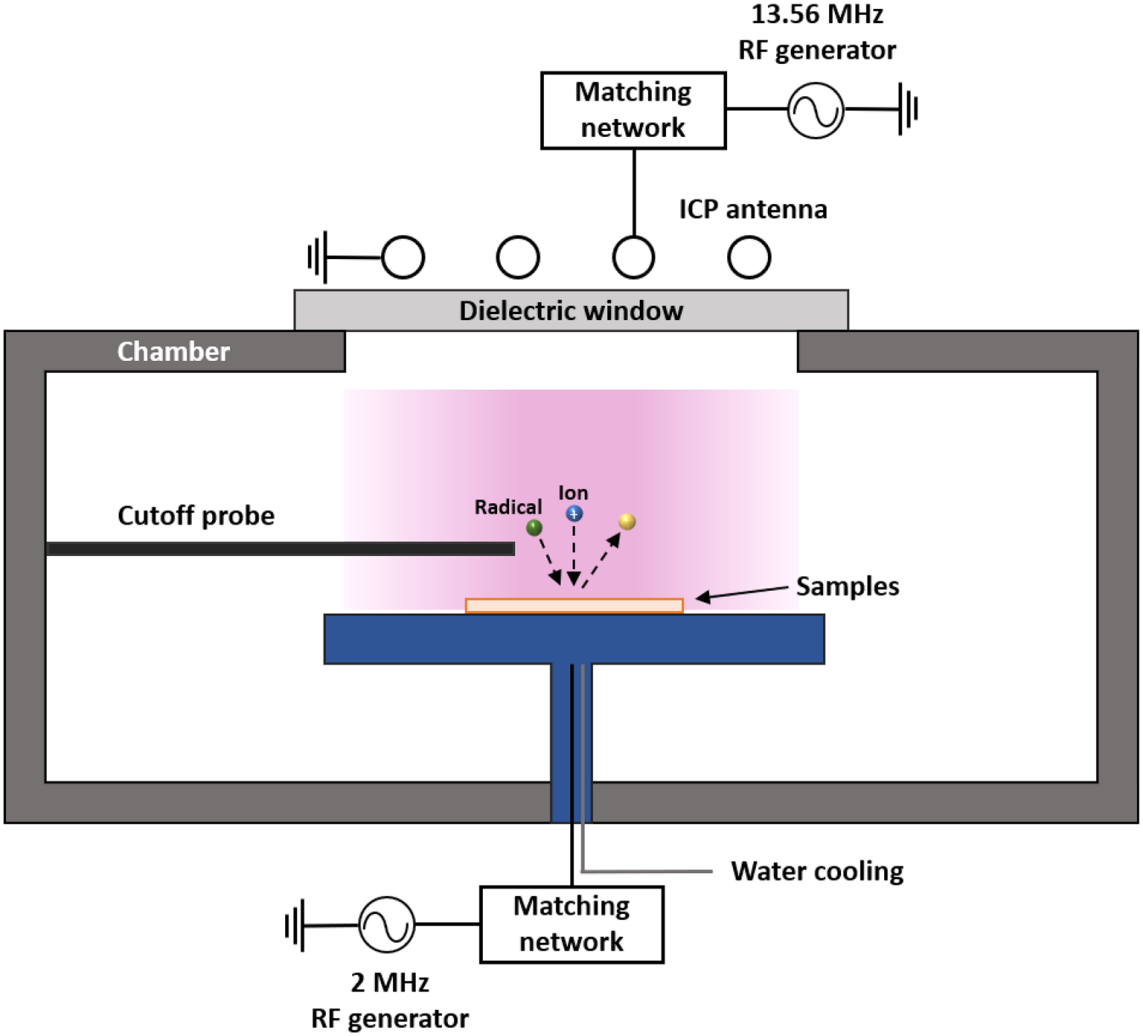
Simple schematic illustration of plasma etching test chamber system.

Plasma etching testing was performed on reference materials including sapphire, Y_2_O_3_ ceramics, and Y_2_O_3_-YAM sintered composite materials, within an aggressive plasma environment. The simple scheme of plasma etching chamber is shown in Fig. 3.

**Figure 4:**
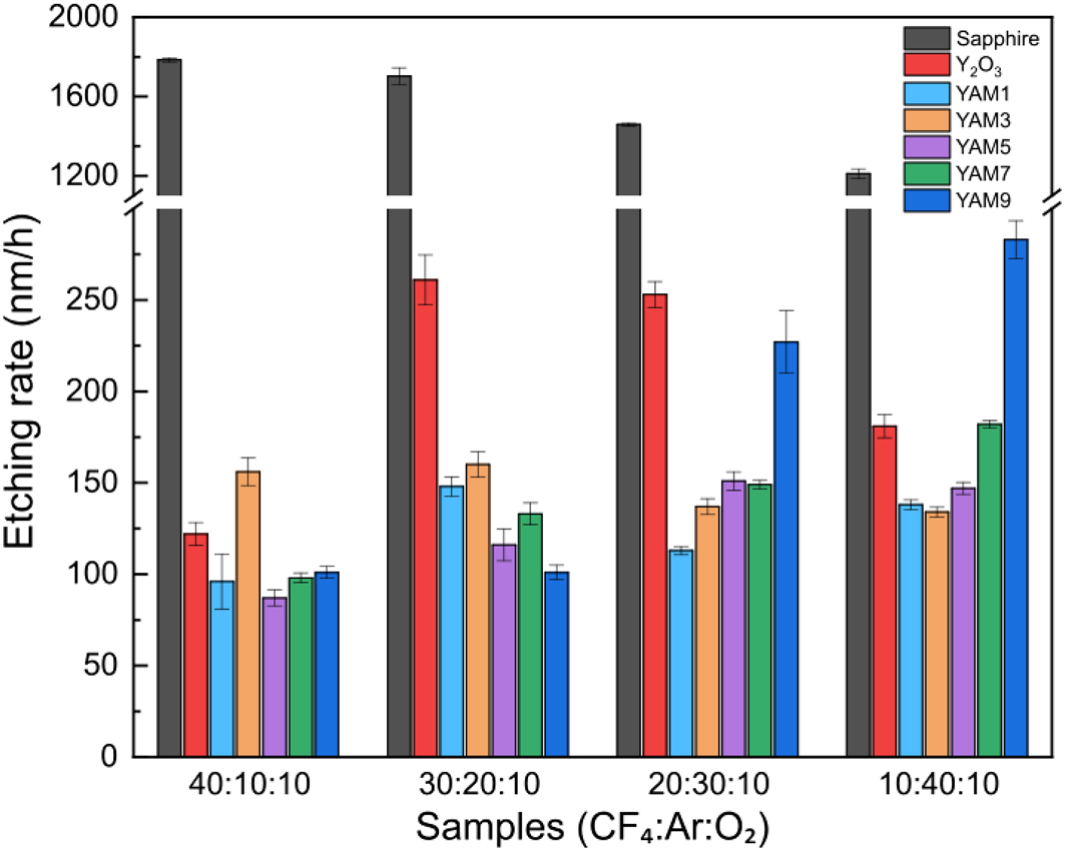
Plasma etching rate of c-axis sapphire, Y_2_O_3_ polycrystalline ceramics and Y_2_O_3_-YAM composites with different volume ratios under a variety of mixed gas ratios between CF_4_, Ar and O_2_ conditions.

Figure 4 illustrates the resultant etching depths of the materials, delineating the impacts of varying plasma gas ratios of CF_4_ to Ar. Sapphire, the reference, showed a fast etching rate of more than 1000 nm/h under all conditions, and the etching depth decreased as the amount of CF_4_ gas decreased because the effect of chemical etching weakened because of the low density of CF_2_ radicals in the gas mixture^42^. For Y_2_O_3_ ceramics, the etching depth increased slightly as the amount of Ar gas increased; it then decreased because the effect of physical etching increased when amount of Ar gas increased and the fluorinated layer generated on the surface of Y_2_O_3_ was easily eliminated^10^. Overall, the Y_2_O_3_-YAM composites exhibited slower etching rates compared to the two reference materials, particularly when materials were subjected to CF_4_:Ar:O_2_ ratios of 20:30:10 and 30:20:10, where simultaneous physicochemical etching was applied. In these conditions, all compositions had inductively coupled plasma-reactive ion etching resistance superior to that of Y_2_O_3_. The overall etching rates of Y_2_O_3_-YAM composites increased with increases in proportion of Ar in the plasma mixture gases. Moreover, the rise in etching rate was more pronounced for higher YAM compositions, notably in the case of the YAM9 composition, the highest YAM contents. As the CF_4_:Ar:O_2_ ratios varied from 40:10:10 to 10:40:10, the etching depth of YAM9 exhibited a nearly threefold increase, increasing from 101 to 283 nm per hour, surpassing the value of Y_2_O_3_. Similarly, the YAM1 composition with 10% YAM content demonstrated an increase in etching depth from 96 to 138 nm. This phenomenon can be attributed to the lower boiling point (1275 °C) of AlF_3_, which formed on the YAM composition surface through its reaction with F^−^ separated from CF_4_ gas. This value is substantially lower than the boiling point of YF_3_ (2230 °C), making it challenging to produce AlF_3_ on the surface. Additionally, compared to YF_3_ (161 eV), the lower binding energy (77 eV) of AlF_3_ renders it more susceptible to removal by physical ion bombardment, thereby influencing the etching rate^43^.

**Figure 5:**
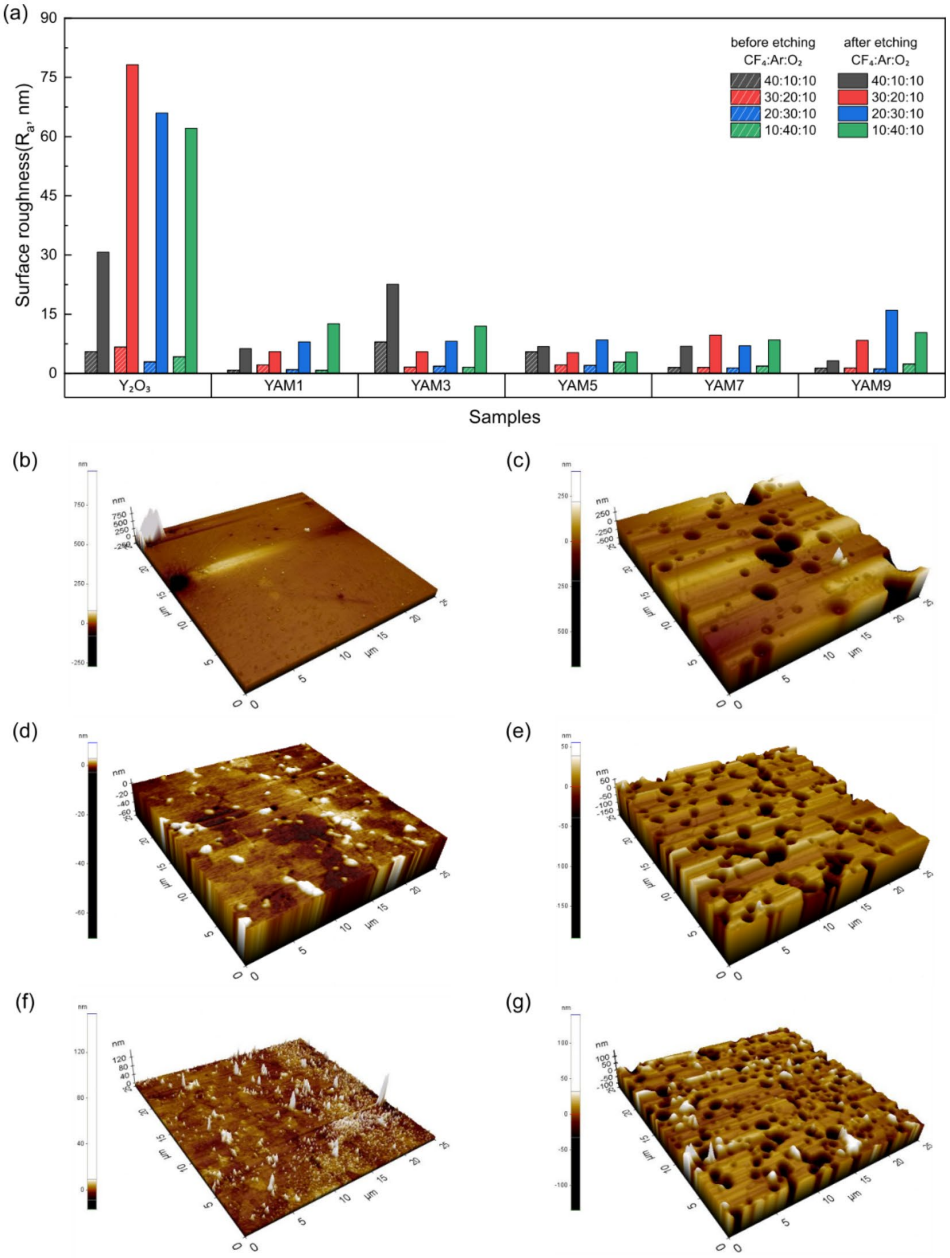
(**a**) Measured surface roughness (R_a_) of Y_2_O_3_ and Y_2_O_3_-YAM composite ceramics at masked and plasma exposed regions with different volume ratios and mixed gas ratios of CF_4_:Ar:O_2_. Representative 3D images of masked and exposed surface morphology were produced by atomic force microscopy (AFM): (**b**) masked and (**c**) etched Y_2_O_3_ polycrystalline ceramics; (**d**) masked and (**e**) exposed Y_2_O_3_-YAM (YAM1) composites; (**f**) masked and (**g**) exposed Y_2_O_3_-YAM (YAM9) composites. CF_4_:Ar:O_2_ gas ratio was 10:40:10.

To further analyze the plasma resistance properties of the composites, surface roughness changes before and after plasma etching, according to the variety of mixed gas ratios and compositions, were investigated. The results are presented in Fig. 5. In Fig. 5a, the Y_2_O_3_ single component ceramic demonstrates a substantial change in surface roughness following plasma etching across all gas composition environments. Especially, in the case of CF_4_:Ar:O_2_ gas with a ratio of 10:40:10, the surface roughness (${R}_{a}$) increased significantly from 4.2 to 62.1 nm (14.8 times). The AFM 3D images in Fig. 5b,c reveal a smooth surface pre-etching, transforming at post-etching into one with large craters, each several micrometers in size. These craters emerged as a consequence of localized and intense impacts on residual pores in the microstructure of the Y_2_O_3_ sintered body by Ar^+^ ion sputtering, resulting in deterioration of the specimen surface^5,6^. The ceramic surface’s irregularities directly affect the release of sputtered neutral atoms and contaminants formation. Elevated irregularities contribute to an increased release of them, potentially giving rise to the generation of particles that are inadequately evacuated^8,44^. Compared to Y_2_O_3_, all Y_2_O_3_-YAM composite had a lower roughness than Y_2_O_3_ after plasma exposure. AFM 3D images before and after etching for these compositions (Fig. 5d–g) distinctly show the discrepancy in plasma etching rates. In cases in which Y_2_O_3_ and YAM compositions were combined at 50:50 volume ratio, changes in surface roughness with plasma etching were minimal. When the CF_4_:Ar:O_2_ gas mixture ratio was 10:40:10, the surface roughness change increased by a mere 1.9 times, from 2.9 to 5.4 nm. This aligns with previous findings on surface roughness change after plasma etching of nanocomposites, suggesting its efficacy in minimizing the formation of large craters. It is well-known that changes of surface roughness are significantly dependent on grain size^26^. Therefore, the positive effect in this study can be attributed to the inhibition of grain growth through a pinning effect and densification achieved by reducing the sintering temperature^25,45^.

**Figure 6:**
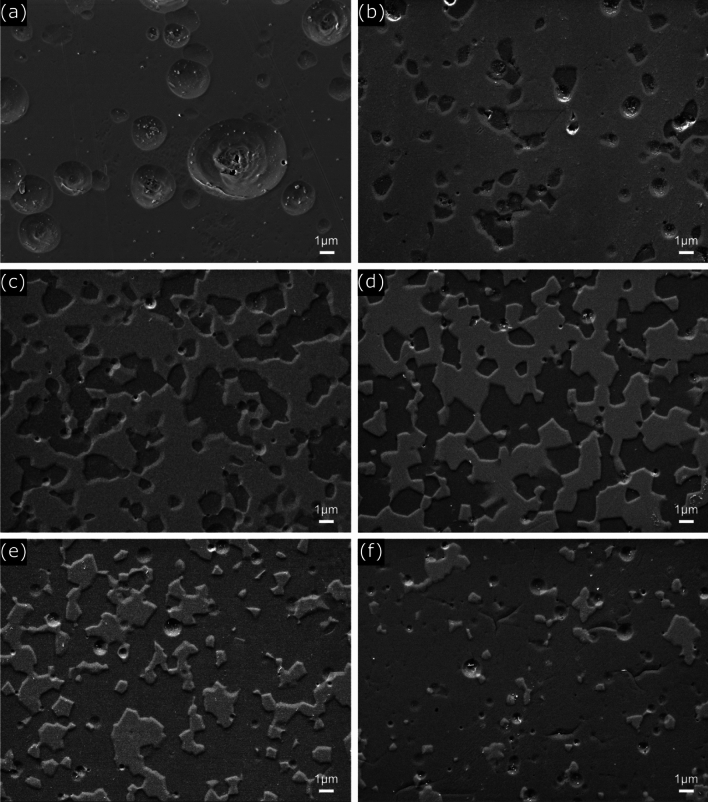
SEM microstructure images of Y_2_O_3_ and Y_2_O_3_-YAM composites with different volume ratios after plasma etching under at 40:10:10 CF_4_:Ar:O_2_ gas ratio; (**a**) Y_2_O_3_ monolith ceramic, (**b**) YAM1, (**c**) YAM3, (**d**), YAM5, (**e**), YAM7, and (**f**) YAM9 composite ceramics.

The SEM images presented in Fig. 6 show microstructures of Y_2_O_3_ and Y_2_O_3_-YAM composites after plasma etching test conducted with CF_4_:Ar:O_2_ gas ratio of 40:10:10. In Fig. 6a, the Y_2_O_3_ polycrystalline ceramic exhibited the development of substantial craters, each several micrometers in size, after plasma etching, covering the entire specimen. As previously mentioned, the emergence of these craters is associated with pronounced etching of micropores within the specimen, particularly in open pores; the resultant large craters can induce noteworthy alterations in surface roughness. Contrastingly, in Fig. 6b–f, for the Y_2_O_3_-YAM composite, dark regions signify YAM composition, while light regions denote Y_2_O_3_ composition, revealing an evident discrepancy in etching rate based on composition. The YAM1 specimen, comprising 10% YAM by volume, underwent more profound etching over a small area, while YAM5, with the same proportion between two compositions, experienced deep etching across nearly half of the area. The lesser bonding energy of Al-O (512 kJ/mol) compared to that of Y–O (685 kJ/mol) underscores the significant role played by the reaction between Al-O bonding and fluorocarbon deposits^46–48^. This reaction results in the formation of AlF_3_ layers, prone to removal via physical attack due to the vulnerability of fluorinated layers to ion sputtering. Consequently, an etching depth differential between the Y_2_O_3_ and YAM compositions arose^15,25,32^. Craters within the Y_2_O_3_-YAM composite microstructure formed, but were very small in size, unlike the results of the Y_2_O_3_ mono-composition. Sizes of craters formed during plasma etching is intricately linked to the ceramic grain size. Hence, amalgamation of varying YAM and Y_2_O_3_ compositions can reduce crater size and minimize changes in surface roughness if grain growth is effectually suppressed^49^. For composition with little solidification at temperatures below the eutectic point, the pinning effect is much more effective at suppressing grain growth; it can dramatically reduce growth; resulting craters rarely form, and if formed, they are very fine^28^. In light of these considerations, a strategic combination of compositions recognized for their robust plasma resistance properties holds promise in effectively diminishing crater size, thereby attenuating changes of surface roughness and reducing contaminant particle generation.

**Figure 7:**
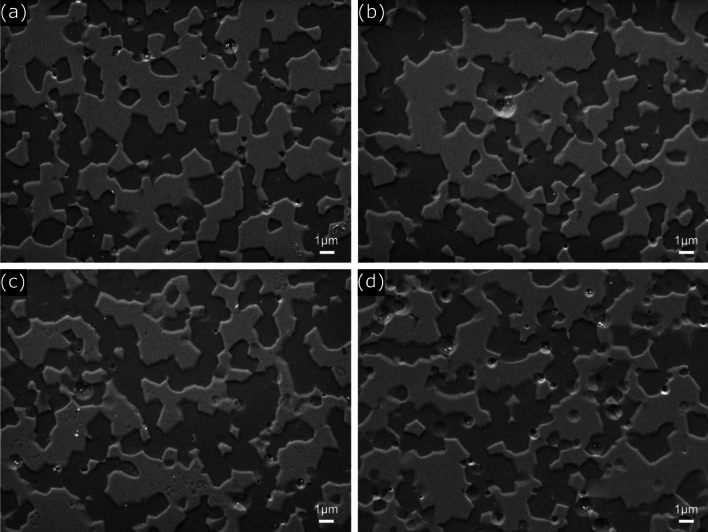
SEM microstructure images of Y_2_O_3_-YAM nanocomposite with 50:50 vol% after plasma etching under different CF_4_:Ar:O_2_ gas ratios of: (**a**) 40:10:10, (**b**) 30:20:10, (**c**) 20:30:10, and (**d**) 10:40:10.

SEM images of the microstructure after plasma etching are shown in Fig. 7; the CF_4_:Ar gas ratio was varied for the YAM5 specimen with a 50:50 volume mixture of Y_2_O_3_ and YAM ceramics. Overall, no significant differences in microstructure were seen with different plasma atmospheres, and there were no changes in composition of preferential etching. As the ratio of Ar gas increased, the number of craters formed on the surface with sizes of 1 $\mu\text{m}$ or less increased, especially when the ratio of CF_4_:Ar:O_2_ was 10:40:10, meaning that there was a very high amount of Ar, as shown in Fig. 7d; in this case, the effect of physical etching increased and more craters were formed. As shown in Fig. 7c, when the ratio of CF_4_:Ar:O_2_ gas was 20:30:10, a large number of irregular nanopores formed on certain Y_2_O_3_ grains^15^.

**Figure 8:**
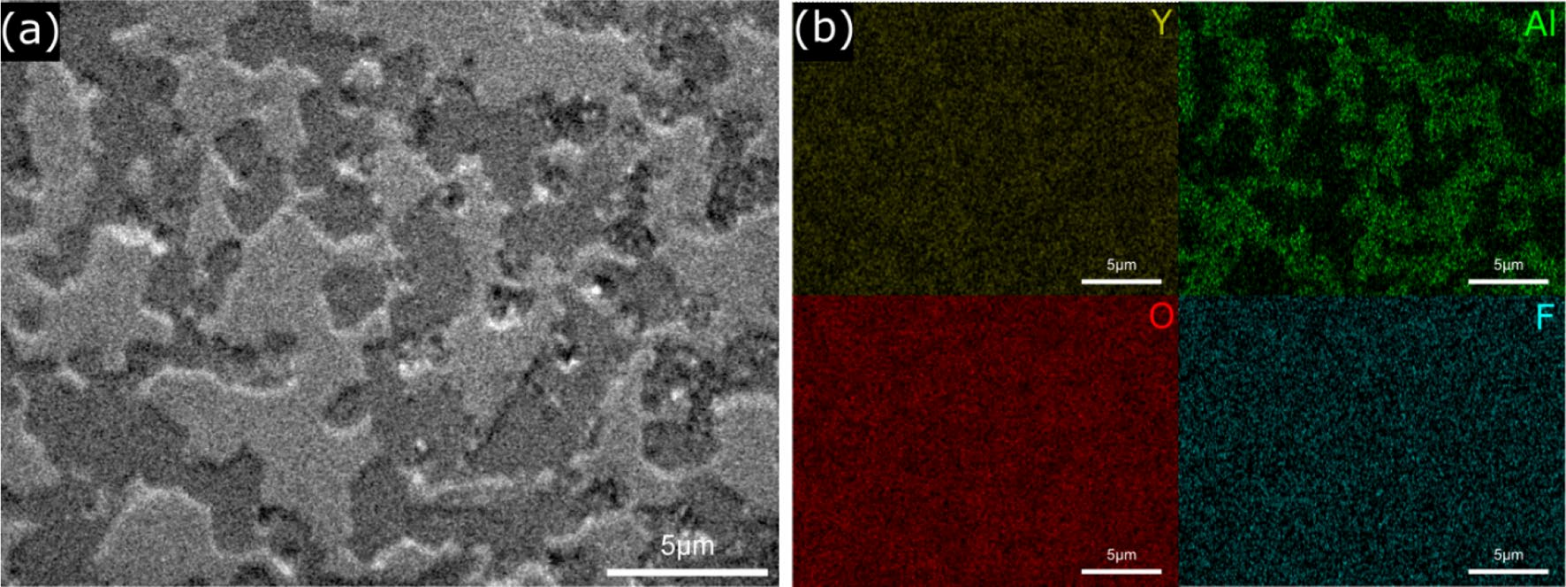
(**a**) SEM microstructure image and (**b**) element mapping result for Y_2_O_3_-YAM (YAM5) composites after plasma etching at 10:40:10 CF_4_:Ar:O_2_ gas ratio.

SEM–EDS analysis of the surface of the YAM5 specimen after etching in CF_4_:Ar:O_2_ gas mixture with ratio of 40:10:10 is shown in Fig. 8. Different compositions exhibited different levels of etching resistance, as shown in Figs. 6 and 8a; the composition with darker colored grains and etched faster by the plasma is YAM composition, and the EDS mapping results confirms that the darker grains are YAM composition, as shown in Fig. 8b. Other compositions of Y_2_O_3_ and F were detected throughout and no significant differences were found. In a recent study, bulk YAG ceramic was found to be etched slightly faster than bulk Y_2_O_3_ ceramic under CF_4_:Ar:O_2_ plasma gas ratios of 40:10:10 and 10:40:10^50^. This study shows that YAM ceramics also etch faster microscopically than Y_2_O_3_ under the same conditions.

**Figure 9:**
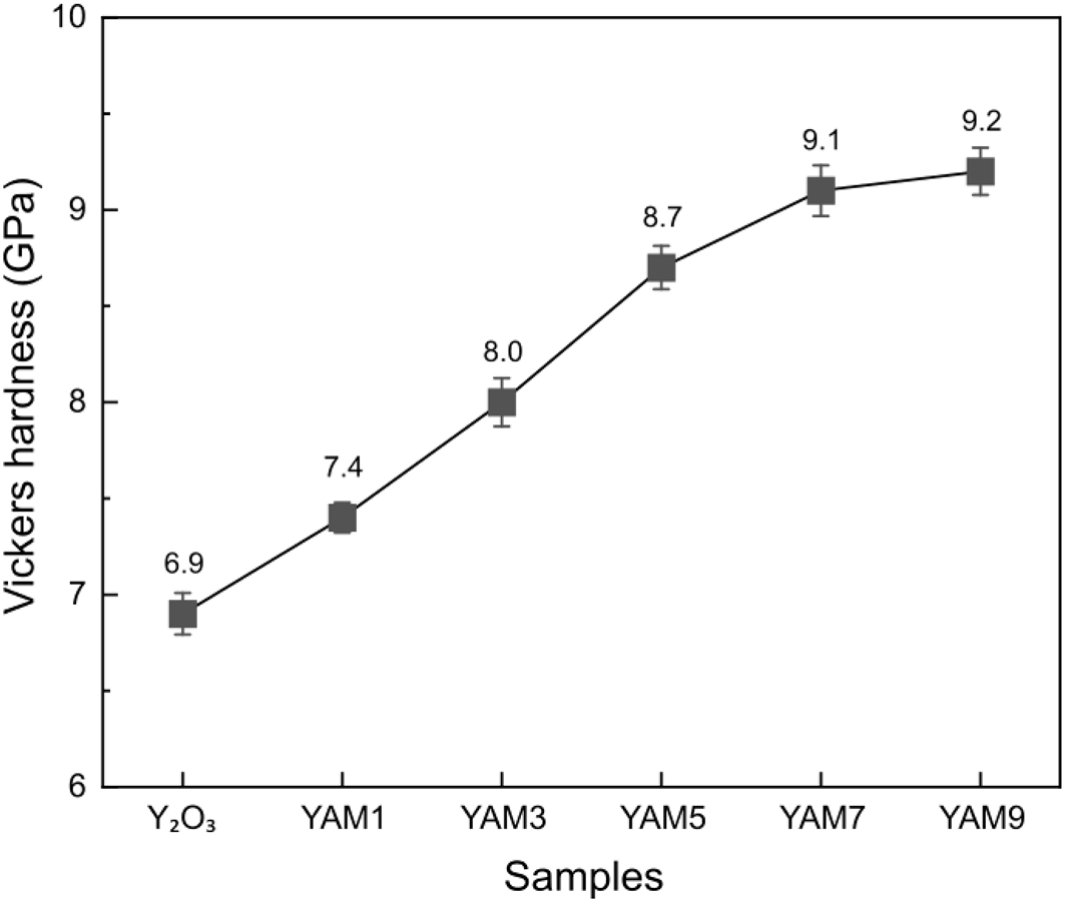
Vickers hardness of Y_2_O_3_ polycrystalline ceramics and sintered Y_2_O_3_-YAM composites with different volume ratios.

Next, the mechanical properties of the Y_2_O_3_ and Y_2_O_3_-YAM composites were evaluated; results of Vickers hardness measurements are shown in Fig. 9. To serve as viable components in a practical semiconductor plasma etching apparatus, materials must have adequate mechanical properties alongside resistance to etching. The Y_2_O_3_ ceramic is acknowledged for its inherently low hardness, typically within a range of 7–8 GPa; a low hardness value of 6.9 GPa was obtained for the specimen in this study^51^. The compromised mechanical properties of Y_2_O_3_ pose impediments to its sustained use of ceramic components, prompting frequent replacement cycles. Therefore, enhancing the mechanical properties without losing plasma etching resistance is imperative. The pure YAM composition has excellent hardness of about 11 GPa^35^. By compounding YAM in Y_2_O_3_, the Vickers hardness improved and, as the proportion of YAM increased from 10 to 90%, the Vickers hardness improved to 9.2 GPa. Consequently, the composite of Y_2_O_3_ and YAM did not deviate much from the Y_2_O_3_ and Al_2_O_3_ series of materials used as in-chamber materials for conventional semiconductor plasma etching processes, making it easy to apply in industry, while minimizing crater formation through grain growth inhibition. The excellent etching resistance, small surface roughness change, and enhanced mechanical properties are expected to enable the material to be used for a longer period and significantly improve the yield of semiconductor production, pushing it beyond the limitation of material components in plasma etching chambers that rely on conventional single composition and coating methods.

## Conclusion

In conclusion, we studied the plasma etching characteristics of Y_2_O_3_-YAM composites and Y_2_O_3_ under harsh environment with different CF_4_:Ar:O_2_ mixed gases ratios. Y_2_O_3_ polycrystalline ceramics showed fast etching rate, large change of surface roughness. In addition, there is formation of large craters at the surface after plasma etching. On the other hand, the Y_2_O_3_-YAM composite showed minimal surface roughness changes, while etching rate increased under the physical etching-dominant environment. Especially, under various gas conditions, composites with 50:50 volume fractions demonstrated superior physicochemical etching resistance compared to Y_2_O_3_ ceramics. The composites also effectively reduced the size of the craters produced on the Y_2_O_3_ surface after plasma attack. Based on these findings, Y_2_O_3_-YAM composites demonstrate remarkable inductively coupled plasma-reactive ion etching resistance in plasma etching conditions, accompanied by a notable decrease in the generation of contaminants. Furthermore, the low hardness, a critical drawback of conventional Y_2_O_3_ ceramics, is significantly enhanced in the composites. Characteristics have significantly improved without departing too far from the candidate materials previously used as components in semiconductor manufacturing process equipment. We contend that this study provides valuable perspectives for improving applications in the semiconductor manufacturing industry.

## Data Availability

The datasets used and/or analysed during the current study available from the corresponding author on reasonable request.
